# Male or Female? The Answer Depends on When You Ask

**DOI:** 10.1371/journal.pbio.0050335

**Published:** 2007-12-27

**Authors:** Helen K Salz

## Abstract

Over 80 years ago, Bridges came to the conclusion that sex in*Drosophila* is determined by the X:A ratio. Doubts about this hypothesis are raised by taking a molecular look at how--and when--sex is determined.

Akey decision in the life of an organism is whether to be male or female. In Drosophila, each cell makes this choice independently of its neighbors such that diploid cells with one X chromosome (XY) are male and those with two chromosomes (XX) are female. In classic experiments carried out more than 80 years ago, Calvin Bridges made two important conclusions about how sex is determined in flies [1–3]. He showed that the Y chromosome is not a factor and suggested that sex is determined not simply by counting X chromosomes, but by calculating the ratio of X chromosomes to the number of sets of autosomes (known as the X:A ratio). The concept that sex is determined by a mechanism that evaluates the number of X chromosomes relative to autosomes was invoked to explain the observation that animals with two X chromosomes and three sets of autosomes (XX; AAA) develop as sexual mosaics rather than females. According to this model, animals with the same number of X chromosomes as autosome sets (ratio of 1) are female, animals with half as many X chromosomes as sets of autosomes are male (ratio of 0.5), and those with an intermediate ratio (XX; AAA; ratio of 0.67) are sexual mosaics. With the finding that haploid cells (X; A; ratio of 1) are female [4,5], the idea that sex is determined by the X:A ratio became enshrined in the literature.

How is sex determined in molecular terms? In diploid cells, the choice to be male or female is made when the activity state of *Sex-lethal* (*Sxl*) is set during early embryogenesis (reviewed in [6–8]). The all or none character of *Sxl* activation depends on the expression levels of four X-encoded proteins: SISA, SCUTE, RUNT, and UNPAIRED. When expression levels reach a threshold, as in XX individuals, the three X-encoded transcription factors—SISA, SCUTE, and RUNT—stimulate transcription by binding the *SxlPe* promoter [9,10]. Activation is then reinforced through the Janus kinase pathway via Unpaired, an X-encoded secreted ligand [11,12]. Once active, the presence of the female-specific SXL RNA-binding protein directs all aspects of female development. In XY animals, the expression levels of the four X-linked gene products produced from the single X chromosome fail to reach the threshold concentration needed to activate *Sxl*, and male development ensues.

The window of opportunity for *Sxl* activation is brief, ending at the cellular blastoderm stage, ~3.0 h after fertilization [6,12–14]. At this time, transcription from the *SxlPe* promoter is shut down, but *Sxl* continues to be transcribed from a different promoter (*SxlPm*) in all embryos. Nevertheless, *Sxl* expression remains female-specific, because the female-specific SXL RNA-binding protein regulates its own expression at the level of splicing (reviewed in [15]). Without the SXL protein (as in XY embryos), *Sxl* transcripts are nonfunctional, because they contain an exon with multiple premature stop codons. In XX embryos, the presence of SXL protein forces the translation-terminating exon to be skipped, thereby generating only protein-encoding mRNAs. The continuous supply of SXL protein produced by this autoregulatory splicing loop reinforces the sex-fate decision made earlier in development by converting the transient X-chromosome dose signal into long-term cellular memory.

How well does the hypothesis that sex is determined by the X:A ratio fit with our molecular understanding of *Sxl* activation? Models incorporating the idea that *Sxl* is the immediate downstream target of the signal emitted from the X:A ratio describe the four X-linked genes required for *Sxl* activation as X chromosome signal elements (XSEs). The collaboration of these proteins to stably activate *Sxl* provides a simple biochemical explanation for the conversion of a 2-fold difference in X chromosome number into an all-or-none response. What distinguishes the X:A ratio model from a simple X chromosome counting mechanism is the prediction that the activity of XSEs is measured against a background of autosomal signal elements (ASEs). In molecular terms, we would expect the ASE gene products to counteract the activating function of the XSEs, making *Sxl* activation impossible unless the XSE gene products “outnumber” the ASE products. While this fits with our understanding of how transcription of major developmental genes is tightly controlled by the competitive balancing of positively and negatively acting regulatory factors, the fact remains that, despite heroic efforts, only a single ASE, encoded by *deadpan*, has been identified [13,16]. Although this is disconcerting (see [6,13,16,17]), the X:A signaling hypothesis has thus far provided the best conceptual framework for explaining why haploid cells with their single X chromosome and an X:A ratio of 1 are female, and the failure to identify additional ASEs has not been considered a fatal flaw—until now.

In work reported in this issue of *PLoS Biology* [18], Erickson and Quintero take a molecular look at haploid embryos and find that *Sxl* is activated not because the X:A ratio equals one, but because the XSE products produced from the single X chromosome are allowed to accumulate longer than their diploid single X chromosome siblings are. *Sxl* activation, like other early events in embryogenesis, happens on a tight time schedule. Immediately after fertilization, wild-type embryos undergo 13 rapid synchronous nuclear divisions in a common cytoplasm, without forming cellular membranes. The transformation from this precellular, or syncytial, embryo to a cellular blastoderm occurs as the division rate slows down, midway through nuclear cycle 14. Embryogenesis in haploids is nearly identical to diploids with one “small” difference—the formation of the cellular blastoderm is delayed by a single division cycle and occurs during nuclear cycle 15 [19].

Erickson and Quintero realized that because sexual fate is determined before cellular blastoderm formation, a mechanism that “reads” an X:A signal predicts that the timing of *SxlPe* activation will be identical in haploids and diploids. By tracking the timing of *scute* and *SxlPe* expression by RNA in situ hybridization, the authors show that in diploids, the build up of this XSE reaches the necessary threshold level for *SxlPe* activation by nuclear cycle 12. The transition to the cellular blastoderm stage is accompanied by the rapid decrease in *scute* mRNA accumulation and the cessation of transcription from *SxlPe*. Haploid embryos undergo the same sequence of events; however, the entire process is delayed until nuclear cycle 14, when the level of *scute* expression reaches the same level as that in diploid cycle 12 embryos. Importantly, whereas these data thoroughly dispel the notion of a static X:A ratio–based signal, they remain entirely consistent with our molecular understanding of *SxlPe* activation and its dependence on reaching threshold concentrations of XSE gene products (Figure 1).

**Figure 1:**
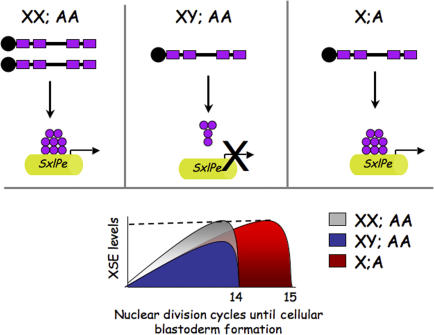
Threshold Response Model for Sex Determination *Sxl* is the direct target of the signals that control sex determination and is activated in response to a threshold concentration of X-linked gene products (in purple). The threshold level of X-linked gene products necessary for transcriptional activation of *Sxl* via the *SxlPe* promoter (indicated by the dotted line)—if it is to occur—must be accomplished by cellular blastoderm, because this developmental milestone is accompanied by a rapid degradation of XSE mRNAs and proteins. In diploids, this level is only reached in XX embryos. In XY embryos, the gene products produced from the single X chromosome do not reach the necessary threshold level, and *Sxl* remains off. In haploids, the cellular blastoderm stage is substantially delayed. Because of this delay, the XSE products produced from the single X chromosome are able to accumulate over a longer period of time, reaching the necessary threshold level for *SxlPe* activation.

Although these studies have released us from the X:A ratio dogma, they leave open the question of why the window of opportunity for *SxlPe* activation is longer in haploids than in diploids. In other words, why are the events leading to *SxlPe* shutdown delayed in haploid embryos? That cellular blastoderm formation is also delayed provides the first hint that these two major developmental milestones are coupled. Further support that *SxlPe* shutdown and cellular blastoderm formation are linked comes from an examination of the *SxlPe* activation time course in XX; AAA embryos. Remarkably, both cellularization and *SxlPe* shutdown happen earlier than in diploids—during cycle 13. Thus, the development of these flies as sexual mosaics can be explained by postulating that *SxlPe* shuts down before some (but not all) cells have reached the concentration of XSEs necessary for *SxlPe* activation. Is cellular blastoderm formation the trigger that initiates *SxlPe* shut down? Cellular blastoderm formation marks the completion of the maternal-to-zygotic transition, a series of reprogramming events that lead to the elimination of maternal mRNAs and the activation of the zygotic genome [20–23]. Perhaps the machinery that choreographs the timing of these cellular events is also responsible for the timely shutdown of *SxlPe*. If so, then we have added a new regulatory dimension to the choice between male and female development.

## Abbreviations

ASE: autosomal signal element
X:A ratio: the ratio of X chromosomes to the number of sets of autosomes
XSE: X chromosome signal element
